# Supplementary material for: PALB2 chromatin recruitment restores homologous recombination in BRCA1-deficient cells depleted of 53BP1
Source: Nat Commun. 2020 Feb 10;11:819. doi: 10.1038/s41467-020-14563-y (PMC7010753; doi:10.1038/s41467-020-14563-y)
Supplement: Supplementary file 2 — Supplementary Information [file 41467_2020_14563_MOESM2_ESM.pdf]

PALB2 chromatin recruitment restores homologous recombination in BRCA1-deficient cells depleted of 53BP1

Belotserkovskaya et al.

Supplementary Materials including:

Supplementary Tables 1-3

Supplementary Figures 1-7

Supplementary table 1. siRNA sequences and references.

Gene name	Sequence	reference
CTRL (Luciferase)	CGUACGCGGAAUACUUCGA	Dharmacon/Horizon
53BP1	GAAGGACGGAGUACUAAUA	{Galanty:2009dp}
CtIP	GCUAAAACAGGAACGAAUC	{Sartori:2007ix}
BRCA1-1	GGAACCUGUCUCCACAAAG	{Bruun:2003ta}
BRCA1-2	GCAGUGAAGAGAUAAAGAA	{Chapman:2013bz}
PALB2-1	GGAAAGAGCCGGUUGUAAA	{Buisson:2010bo}
PALB2-5 (3'UTR)	GGAGAAUAUCTGAAUGACA	{Zhang:2009el}
BRCA2	AACUGAGCAAGCCUCAGUCAA	{Fan:2006ih}
REV7_1	AGAUCCAGGUCAUCAAGGA	{Dev:2018ir}
RIF1_1	AGACUUGUCUCAGAUAUAA	{Dev:2018ir}
RNF168	GGCGAAGAGCGAUGGAAGA	{Doil:2009ho}
SHLD2_1	GCGUGUGACAUAAGAGAUU	{Dev:2018ir}
SHLD2_2	GCUUUCAGUUCUUUGGAAU	{Dev:2018ir}

Supplementary Table 2. List of primers used to generate sgRNAs for Cas9^{D10A}-mediated gene knock-outs and endogenous tagging.

Gene name; exon #	gRNA_Forward	gRNA_Reverse
PALB2; exon 1	AGGAGAAGGAAAAGGTGCCG	CGGCACCTTTTCCTTCTCCT
	GGGAGGCTCGTCCATCGGGC	GCCCGATGGACGAGCCTCCC
53BP1; exon 10	GCAGTCCCACAGAGCAAGAA	TTCTTGCTCTGTGGGACTGC
	GAACGATAAAAGGAGTAGAT	ATCTACTCCTTTTATCGTTC
BRCA1; exon 9	CAGTTTGGATTCTGCAAAAA	TTTTTGCAGAATCCAAACTG
	TTCATCCCTGGTTCCTTGAG	CTCAAGGAACCAGGGATGAA
RNF168; exon 1	CACTGGCACTCGGACAGCGA	TCGCTGTCCGAGTGCCAGTG
	GATCTGCATGGAAATCCTCG	CGAGGATTTCCATGCAGATC

Supplementary Table 3. List of antibodies used in this study

Antigen	Raised in/type: poly/monoclonal	Source, clone information	Dilution for IB	Dilution for IF
53BP1	Rabbit/poly	Novus Biologicals, NB100-304	1:2000	1:500
ATM	Rabbit/mono	Abcam, [Y170], ab32420	1:2000	
BRCA1	Mouse/mono	Santa Cruz Biotechnology, D9	1:200	1:100
BRCA2	Mouse/mono	Calbiochem/Merck, OP95	1:500	
CtIP	Mouse/mono	Richard Baer, clone 14-1	1:50	
Cyclin A	Mouse/mono	BD Biosciences, 611268	1:1000	1:100
GFP	Mouse/mono	Roche, 7.1+13.1	1:1000	
GFP	Rabbit/poly	Life Technologies, 11122		1:1000
H2AX	Rabbit/poly	Abcam, ab11175	1:5000	
H2B	Rabbit/poly	Abcam, ab1790	1:5000	
H3	Rabbit/poly	Abcam, ab1791	1:10000	
H4	Rabbit/mono	CST, 13919	1:1000	
H2A	Rabbit/poly	Abcam, ab18255	1:1000	
6xHis	mouse/mono	Genscript/ A00186	1:20000	
MBP	mouse/mono	NEB/ E8032S	1:50000	
MBP	Rabbit/poly	Sigma Aldrich, MBP-17	1:50000	
MRG15	Rabbit/mono	Cell Signaling, D2Y4J, 14098	1:1000	
NBS1	Rabbit/poly	Abcam, ab23996	1:1000	
PALB2	Rabbit/poly	Gift of Bing Xia, [25]	1:500	1:100
RAD50	Mouse/mono	Abcam, [13B3/2C6], ab89	1:1000	
RAD51	Rabbit/poly	Santa Cruz Biotechnology, H-92	1:500	1:100
RNF168	Rabbit/poly	Millipore, ABE367	1:1000	
RNF169	Rabbit/poly	Abcam, ab87711	1:1000	
RPA32	Mouse/mono	Abcam, [9H8], ab2175		1:250
RPA32	Mouse/mono	Mouse hybridoma, Shiloh lab	1:20	
β-tubulin	Mouse/mono	Sigma Aldrich, T9026	$1:10^4$	

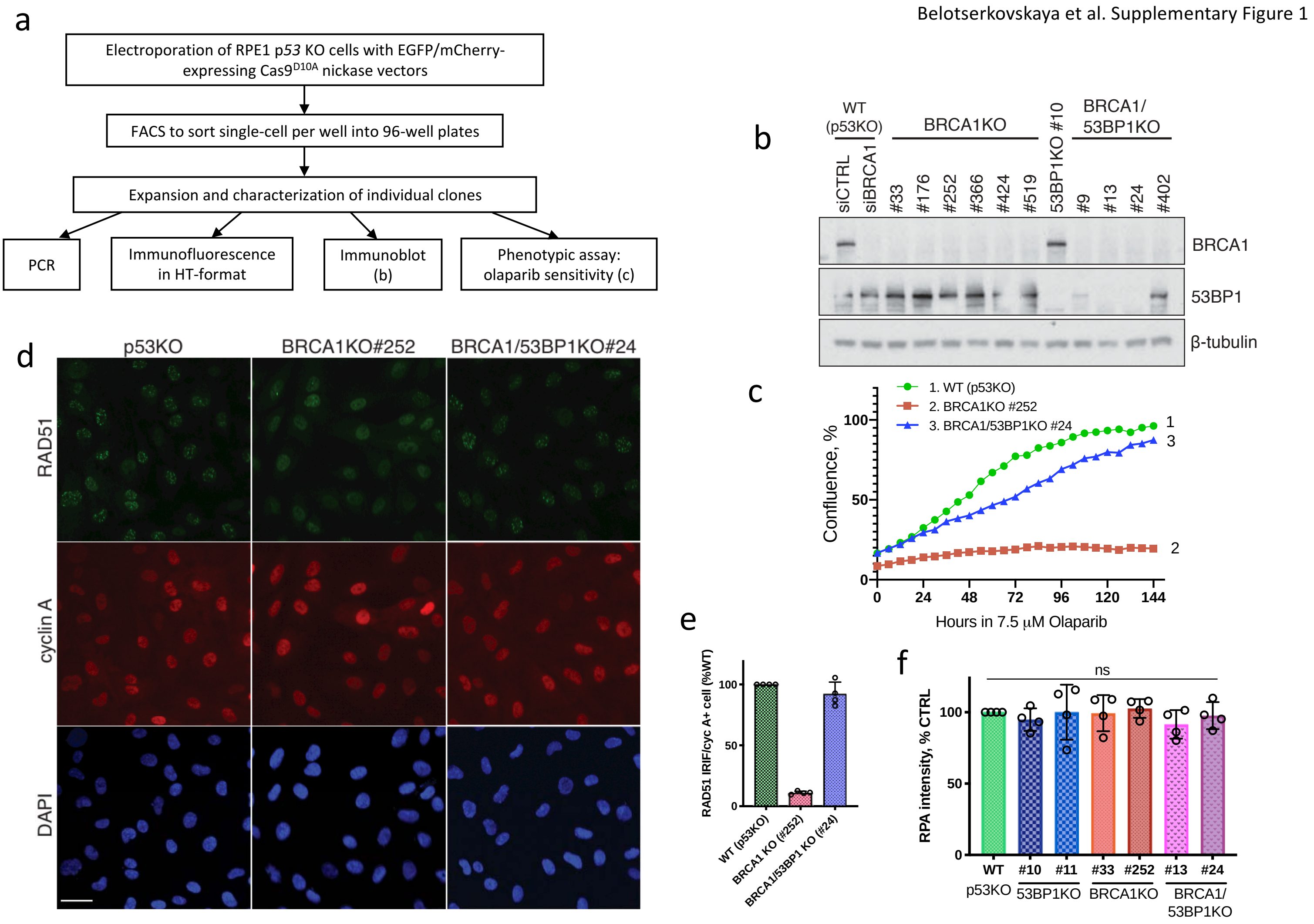

Supplementary Figure 1. CRISPR/Cas9-mediated gene knock outs of *BRCA1* and/or *53BP1*.
(a) Diagram of the workflow for generation and characterization of CRISPR-Cas9-mediated gene knock-outs of either *BRCA1* alone or in combination with *53BP1*. **(b)** Immunoblot of whole cell lysates prepared from cell lines isolated as single-cell clones following CRISPR-Cas9-mediated gene targeting of *BRCA1* or *BRCA1/53BP1* genes in RPE1 *p53*KO cells. Clones selected for immunoblot analyses had all been tested by PCR-amplification across the targeted site and showed changes in mobility of the PCR products, compared to the intact genomic locus (not shown). siRNA-mediated depletion of BRCA1 and 53BP1 was used to control for the protein presence. **(c)** Functional characterization of *BRCA1* and *BRCA1/53BP1* KO clones by assessing their sensitivity to PARP1 inhibitor olaparib. Indicated RPE1 *p53* KO-derived cell lines were incubated with/without olaparib and subject to time-lapse imaging in IncuCyte. Cell culture confluence over time was used as a metric to determine cellular sensitivity to olaparib. Area under curve is shown next to the cell confluence graph. The experiment was performed once as part of the screening process, the confluence data was acquired in two technical replicas, thus the data were not subjected to statistical analysis. **(d)** Representative images, acquired on OPERA Phoenix HT microscope, of cyclin A- and RAD51-stained RPE1 KO cells fixed 6 hours after being treated with 6Gy of IR. Scale bar, 50 μm **(e)** Quantification of RAD51 IRIF in RPE1 KO cell lines. **(f)** RPA nuclear intensity in RPE1 *p53* KO-based cell lines with *BRCA1* or *BRCA1/53BP1* genes knocked-out by CRISPR/Cas9, quantified using flow cytometry-based approach. Prior to fixation and staining, cells were treated with 1 μM CPT for 1 hour.

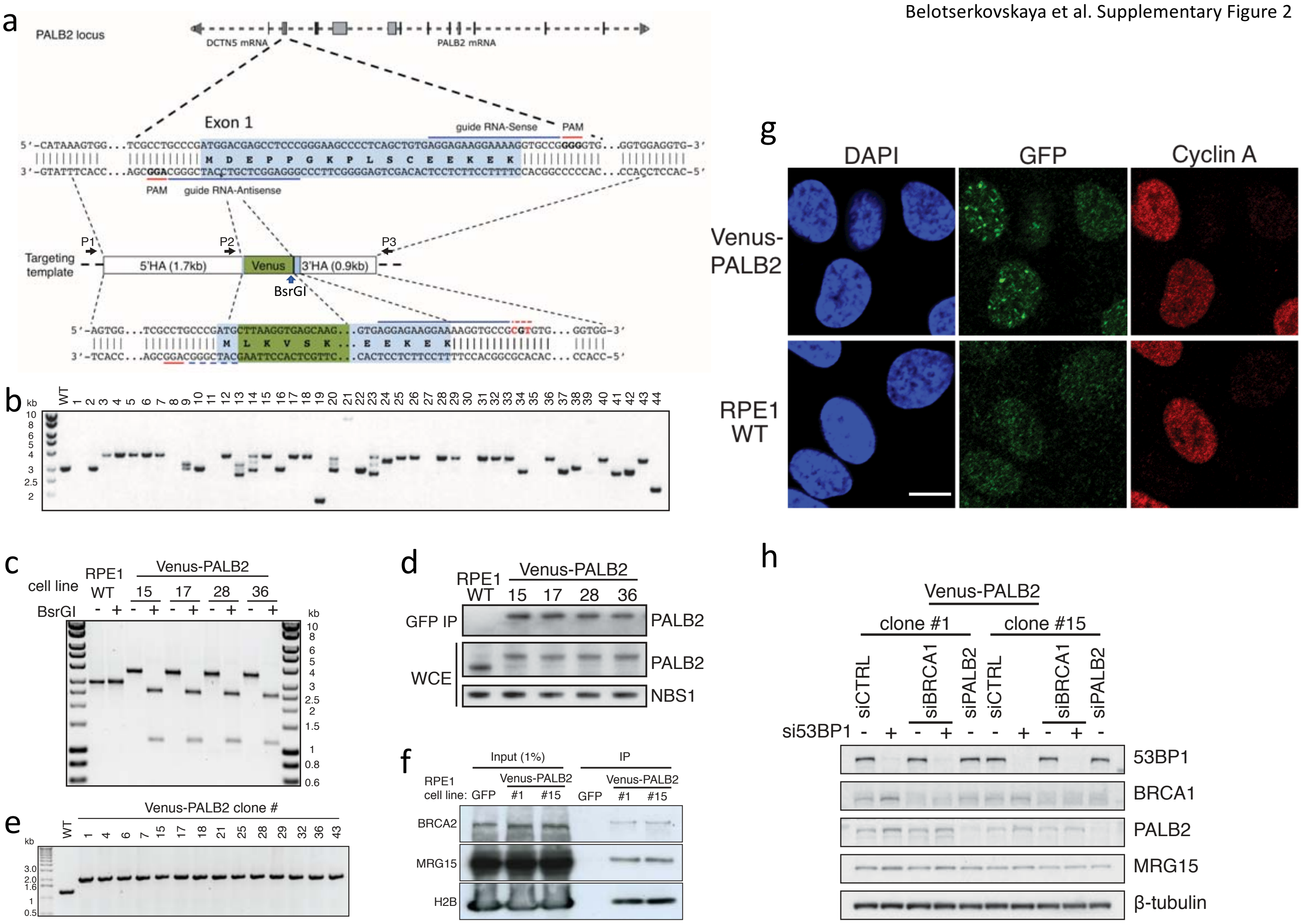

Supplementary Figure 2. Endogenous gene tagging of *PALB2* with Venus tag.

(a) Schematic overview of the N-terminal Venus tagging of PALB2. The antisense sgRNA target sequence is destroyed upon successful integration of Venus. To prevent the sense sgRNA from nicking both endogenous and template plasmid DNA, two mutations (highlighted in red) were introduced in the PAM sequence. PCR primers P1, P2, P3 and BsrGI restriction site used in the ensuing screens for Venus-tag integration are indicated. **(b)** Genotypic screen of surviving clones. DNA fragments generated by PCR using primers P1 and P3 (as shown in **a**), which anneal outside the homology arms and amplify across the Venus integration site, were resolved on agarose gel to indicate Venus insertion (slower migrating band at ~3.5 kb). **(c)** Four clones from **b** were digested with BsrGI (as indicated in **a**), which recognizes the unique restriction site present in Venus sequence. **(d)** Immunoprecipitation (IP) of Venus-PALB2 from whole cell extracts of clones used in **c** by means of GFP-trap agarose beads. Antibody detection of both IP'ed samples (top strip) and input (two bottom strips) as indicated. **(e)** Confirmation of the correct Venus-tag integration in the clones tested positive either in initial PCR screen (presented in **b**) or in high-throughput imaging screen (not shown). As it was not possible to amplify the 3.5 kb DNA product from gDNA of clone #1 using primer pair P1 and P3 (see **b**), PCR products were generated using primers P2 and P3 (as shown in **a**) to yield a ~1.7 kb fragment. P2 anneals within the tagging cassette just upstream of the Venus ORF, while P3 anneals outside the right homology arm in the body of *PALB2* gene, ~1 kb downstream of the start codon. **(f)** Co-IP of endogenously-tagged Venus-PALB2 protein from clones #1 and #15 with its constitutive interaction partners BRCA2 and MRG15 to further confirm that clones #1 and #15, which were used in most experiments, contain functional Venus-PALB2. Anti-GFP IP from whole cell lysates of RPE1 cells with constitutive expression of GFP protein was used as a control. GFP-trap agarose beads were used as in **d**. **(g)** Visualization of Venus-PALB2 IRIF in cyclin A-positive cells exposed to 6 Gy of IR and fixed and stained 6 hours later. Anti-GFP antibody was used to enhance Venus-PALB2 IRIF immunofluorescence. Images were acquired on Olympus Upright confocal microscope. Scale bar, 10 µm. **(h)** Immunoblot of RPE1 Venus-PALB2 clones #1 and #15 depleted for indicated proteins used for the experiment described in Fig. 2a,b.

a

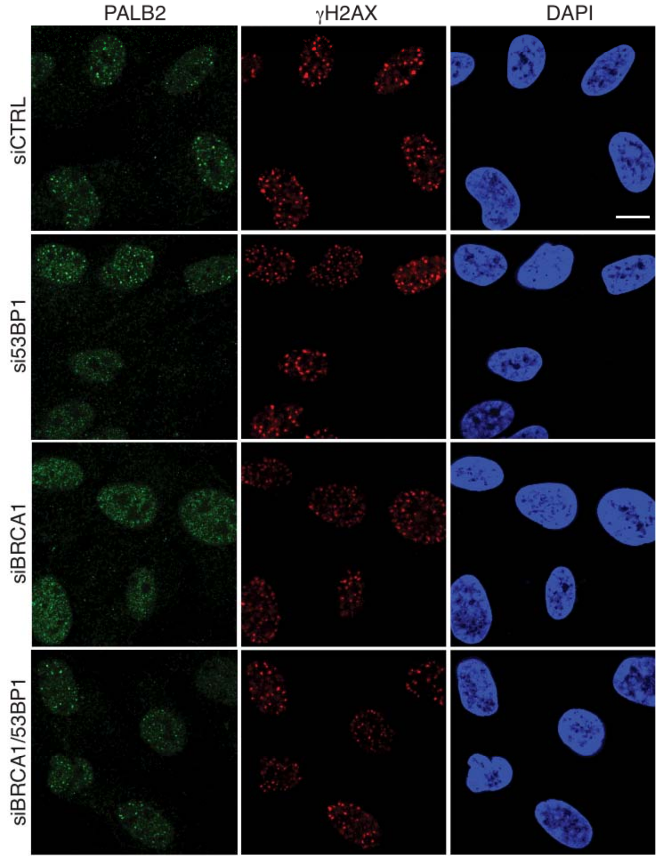

b

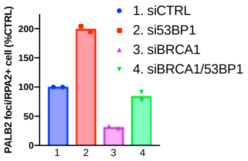

siRNA	experiment 1			experiment 2		
	# cells	PALB2 foci/cell	% CTRL	# cells	PALB2 foci/cell	% CTRL
siLuc	1431	3.250045	100	1522	2.647545	100
si53BP1	1353	6.633515	204.105328	1252	5.15555	194.729457
siBRCA1	891	1.06238	32.6881628	1377	0.770063	29.0859268
si53BP1+siBRCA1	1408	2.500345	76.9326271	1582	2.41796	91.3283816

c

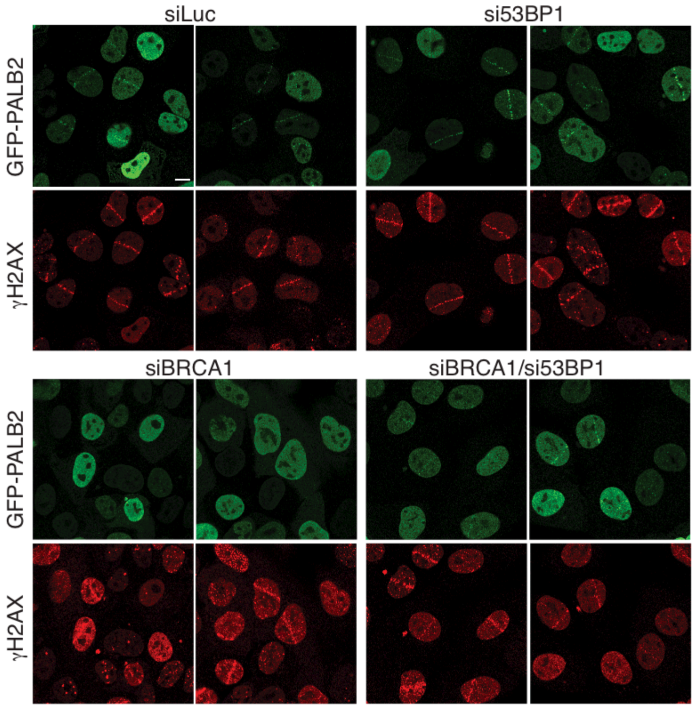

d

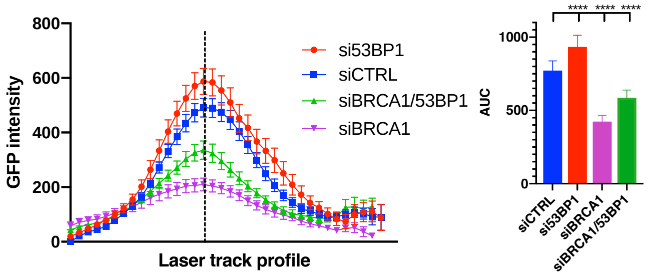

Supplementary Figure 3. 53BP1 depletion rescues PALB2 recruitment to DSBs in BRCA1-deficient cells
(a) Representative images acquired on an Olympus Upright confocal microscope of RPE1 cells siRNA-depleted for BRCA1, 53BP1 or both, exposed to 6Gy IR and, 6 hours later, fixed and stained with antibodies to the endogenous PALB2 protein and γH2AX signal. Scale bar, 10 µm. **(b)** Quantification of PALB2 focus numbers in RPA2-positive cells relative to siCTRL-depleted cells. The images were acquired on an OPERA Phoenix HT microscope in two independent experiments with at least 890 cells analysed per experiment. Cell numbers and PALB2 focus numbers, actual and normalised, are in the table next to the graph. **(c)** 53BP1 co-depletion rescues GFP-PALB2 accrual at DSBs induced by the laser micro-irradiation in BRCA1-depleted cells. Cell line: U2OS-TR with stable Tet-inducible GFP-PALB2. Scale bar, 10 µm. **(d)** Quantification of GFP-PALB2 intensities across the laser tracks: left graph shows GFP intensity profiles; right graph represents AUC calculated based on the profiles. The profiles were generated by subtracting background fluorescence outside of the laser line from the rest of the measurements. For each siRNA-depleted sample, 30-50 profiles were analysed.

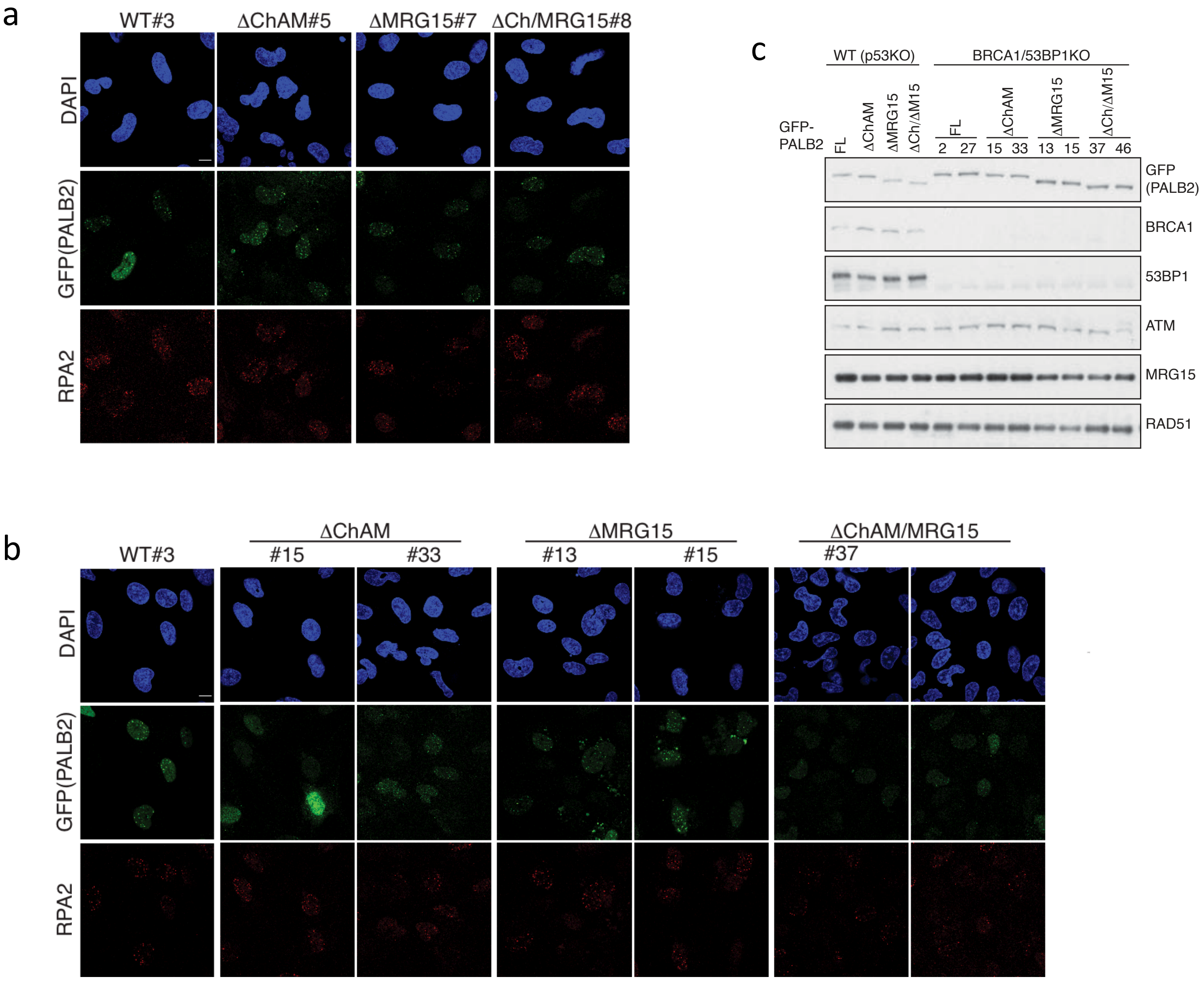

Supplementary Figure 4. ChAM of PALB2 is important for PALB2 recruitment to DSBs in BRCA1/53BP1 deficient cells.
(a-b) Additional representative images of IRIF formed by indicated doxycycline-inducible GFP-PALB2 derivatives expressed in RPE1 *p53* KO (Fig. 3b) or RPE1 *p53/BRCA1/53BP1* KO (Fig. 3d) cells that had been irradiated with 6 Gy of IR and fixed/stained 6 hours later. Violin plots depict quantification of the integrated intensity of GFP-PALB2 IRIF in RPA-positive cells following exposure to 6 Gy of IR. Scale bar, 10 µm. **(c)** Immunoblot of GFP-PALB2 derivatives expressed in RPE1 p53 KO cells after depletion of endogenous PALB2 with 3’UTR-targeting siRNA followed by addition of doxycycline.

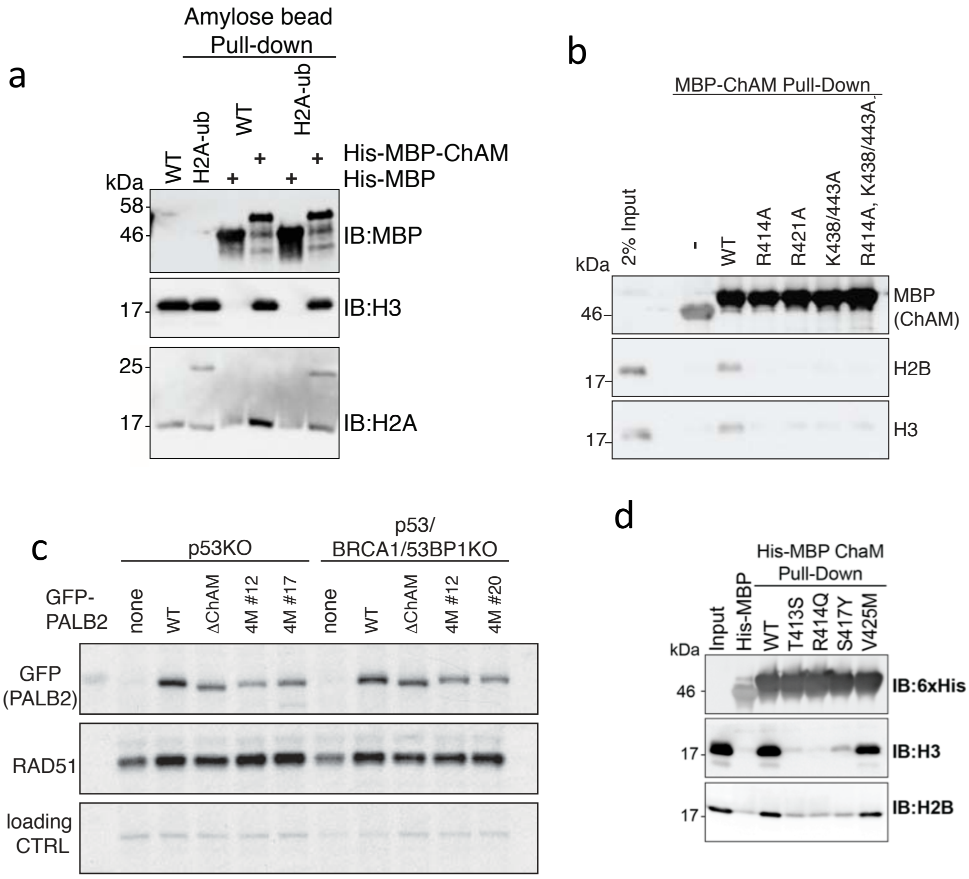

Supplementary Figure 5. Interaction between ChAM of PALB2 and NCP is independent of H2A K15ub but involves contact with the acidic patch

(a) Pull-down assay using purified components. 6xHis-MBP-tagged PALB2 ChAM domain or 6xHis-MBP immobilised on amylose affinity beads, incubated with recombinant nucleosome core particles, with or without chemical ubiquitylation at H2A lysine 15. **(b)** Pull-down assay using MBP-tagged PALB2 ChAM WT and indicated point mutants of evolutionarily conserved basic residues, incubated with recombinant nucleosomes. **(c)** Immunoblot of doxycycline-inducible GFP- $PALB2^{WT,\,-}$ $PALB2^{\Delta ChAM}$ or $PALB2^{4M}$ (PALB2 protein with 4 point mutations in ChAM domain) proteins expressed in RPE1 *p53* KO and *p53/BRCA1/53BP1* KO cells used in clonogenic survivals presented in (F) and (G), respectively. **(d)** Pull-down assay using immobilised His-MBP-ChAM variants containing reported point mutations found in cancer patients. His-MBP included as a negative control.

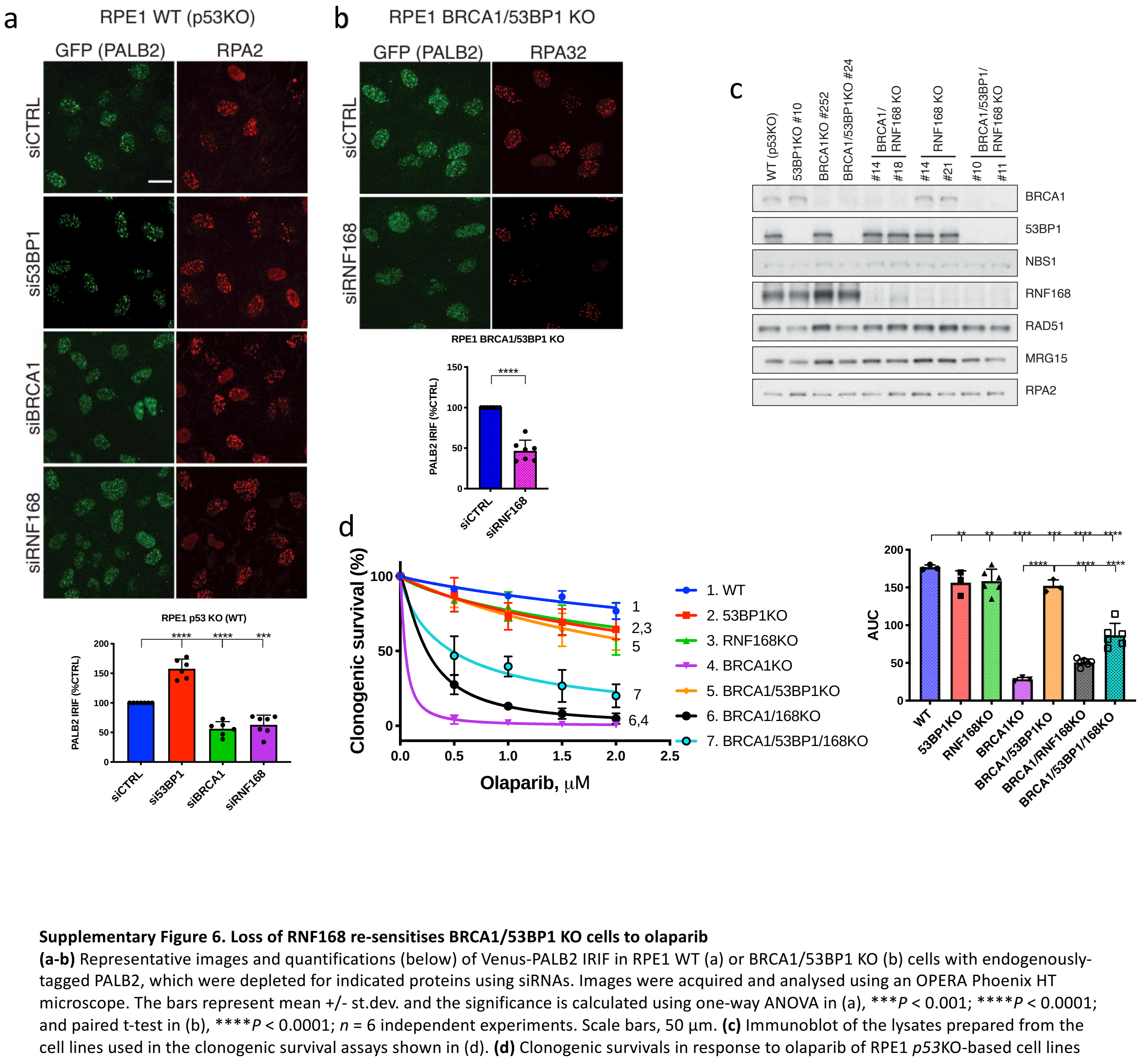

Supplementary Figure 6. Loss of RNF168 re-sensitises BRCA1/53BP1 KO cells to olaparib

(a-b) Representative images and quantifications (below) of Venus-PALB2 IRIF in RPE1 WT (a) or BRCA1/53BP1 KO (b) cells with endogenously-tagged PALB2, which were depleted for indicated proteins using siRNAs. Images were acquired and analysed using an OPERA Phoenix HT microscope. The bars represent mean +/- st.dev. and the significance is calculated using one-way ANOVA in (a), ***$P < 0.001$; ****$P < 0.0001$; and paired t-test in (b), ****$P < 0.0001$; $n = 6$ independent experiments. Scale bars, 50 µm. **(c)** Immunoblot of the lysates prepared from the cell lines used in the clonogenic survival assays shown in (d). **(d)** Clonogenic survivals in response to olaparib of RPE1 *p53*KO-based cell lines with additional KO's of *BRCA1*, *53BP1* and *RNF168* genes. The AUC bars represent mean +/- s.e.m.; one-way ANOVA; **$P < 0.01$, ***$P < 0.001$; ****$P < 0.0001$, ns, not significant ($P \geq 0.05$); $n = 3$ independent experiments.

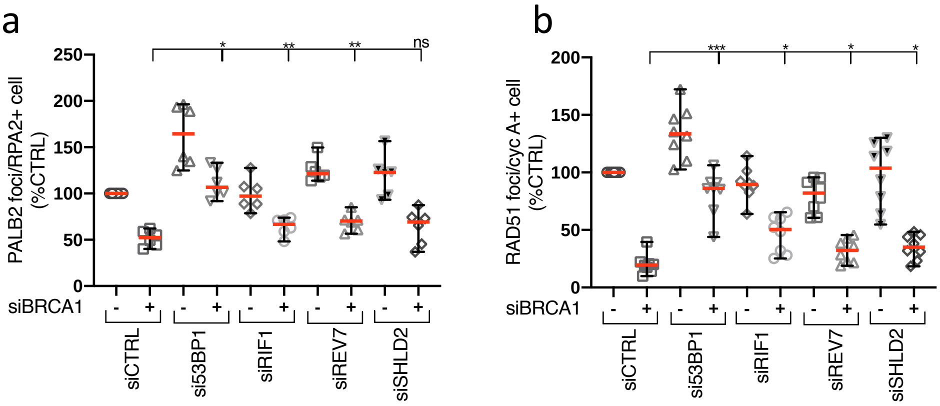

Supplementary Figure 7. Effects of co-depletion of 53BP1-Shieldin axis components on PALB2 and RAD51 focus formation in BRCA1-depleted cells.
a Quantification of Venus-PALB2 IRIF in RPA2-positive cells siRNA-depleted for indicated proteins, exposed to 5Gy of IR and processed for IF 6 hours later. The bars represent means and range from 6 independent experiments; one-way ANOVA; *$P < 0.05$, **$P < 0.01$; ns, not significant ($P \geq 0.05$). **b** Quantification of RAD51 IRIF in cyclin A-positive cells. Treatment and analysis were as in (**a**), except that the data were acquired in 8 independent experiments.

Supplementary References:

1. Galanty, Y. *et al.* Mammalian SUMO E3-ligases PIAS1 and PIAS4 promote responses to DNA double-strand breaks. *Nature* **462,** 935–939 (2009).
2. Sartori, A. A. *et al.* Human CtIP promotes DNA end resection. *Nature* **450,** 509–514 (2007).
3. Bruun, D. *et al.* siRNA depletion of BRCA1, but not BRCA2, causes increased genome instability in Fanconi anemia cells. *DNA Repair (Amst.)* **2,** 1007–1013 (2003).
4. Chapman, J. R. *et al.* RIF1 is essential for 53BP1-dependent nonhomologous end joining and suppression of DNA double-strand break resection. *Mol. Cell* **49,** 858–871 (2013).
5. Buisson, R. *et al.* Cooperation of breast cancer proteins PALB2 and piccolo BRCA2 in stimulating homologous recombination. *Nat. Struct. Mol. Biol.* **17,** 1247–1254 (2010).
6. Zhang, F., Fan, Q., Ren, K. & Andreassen, P. R. PALB2 functionally connects the breast cancer susceptibility proteins BRCA1 and BRCA2. *Mol. Cancer Res.* **7,** 1110–1118 (2009).
7. Fan, S., Meng, Q., Auborn, K., Carter, T. & Rosen, E. M. BRCA1 and BRCA2 as molecular targets for phytochemicals indole-3-carbinol and genistein in breast and prostate cancer cells. *Br. J. Cancer* **94,** 407–426 (2006).
8. Dev, H. *et al.* Shieldin complex promotes DNA end-joining and counters homologous recombination in BRCA1-null cells. *Nat. Cell Biol.* **20,** 954–965 (2018).
9. Doil, C. *et al.* RNF168 binds and amplifies ubiquitin conjugates on damaged chromosomes to allow accumulation of repair proteins. *Cell* **136,** 435–446 (2009).
10. Rappold, I., Iwabuchi, K., Date, T. & Chen, J. Tumor suppressor p53 binding protein 1 (53BP1) is involved in DNA damage-signaling pathways. *J. Cell Biol.* **153,** 613–620 (2001).
11. Xia, B. *et al.* Control of BRCA2 cellular and clinical functions by a nuclear partner, PALB2. *Mol. Cell* **22,** 719–729 (2006).
