# Supplementary material for: PALB2 chromatin recruitment restores homologous recombination in BRCA1-deficient cells depleted of 53BP1
Source: Nat Commun. 2020 Feb 10;11:819. doi: 10.1038/s41467-020-14563-y (PMC7010753; doi:10.1038/s41467-020-14563-y)
Supplement: Supplementary file 3 — Description of Additional Supplementary Files [file 41467_2020_14563_MOESM3_ESM.pdf]

**Description of Additional Supplementary Files**

File Name: Supplementary Movie 1

Description: Visualisation of PALB2 and RPA2 IRIF by 3D-SIM in mock-depleted cells. 3D reconstruction of 3D-SIM acquired z-series of cells presented in Fig. 2c, transfected with siCTRL. Larger RPA2 foci (in red) co-localise with smaller PALB2 foci (in green).

File Name: Supplementary Movie 2

Description: Visualisation of PALB2 and RPA2 IRIF by 3D-SIM in 53BP1-depleted cells. 3D reconstruction of 3D-SIM acquired z-series of cells presented in Fig. 2c, transfected with si53BP1. Larger RPA2 foci (in red) co-localise with smaller PALB2 foci (in green). 53BP1 depletion results in enhanced PALB2 association with RPA2 foci.

File Name: Supplementary Movie 3

Description: Visualisation of PALB2 and RPA2 IRIF by 3D-SIM in BRCA1-depleted cells. 3D reconstruction of 3D-SIM acquired z-series of cells presented in Fig. 2c, transfected with siBRCA1. Compared to mock- and 53BP1-depleted cells, where larger RPA2 foci (in red) co-localise with smaller PALB2 foci (in green), upon BRCA1 depletion, the association between PALB2 and RPA2 foci is drastically reduced.
