# Supplementary material for: PALB2 chromatin recruitment restores homologous recombination in BRCA1-deficient cells depleted of 53BP1
Source: Nat Commun. 2020 Feb 10;11:819. doi: 10.1038/s41467-020-14563-y (PMC7010753; doi:10.1038/s41467-020-14563-y)
Supplement: Supplementary file 7 — Reporting Summary [file 41467_2020_14563_MOESM7_ESM.pdf]

nature research

Corresponding author(s): Rimma Belotserkovskaya, Marcus Wilson, Stephen Jackson

Last updated by author(s): Jan 7, 2020

Reporting Summary

Nature Research wishes to improve the reproducibility of the work that we publish. This form provides structure for consistency and transparency in reporting. For further information on Nature Research policies, see Authors & Referees and the Editorial Policy Checklist.

Statistics

For all statistical analyses, confirm that the following items are present in the figure legend, table legend, main text, or Methods section.

n/a	Confirmed	
☐	☒	The exact sample size (n) for each experimental group/condition, given as a discrete number and unit of measurement
☐	☒	A statement on whether measurements were taken from distinct samples or whether the same sample was measured repeatedly
☐	☒	The statistical test(s) used AND whether they are one- or two-sided<br>*Only common tests should be described solely by name; describe more complex techniques in the Methods section.*
☐	☒	A description of all covariates tested
☐	☒	A description of any assumptions or corrections, such as tests of normality and adjustment for multiple comparisons
☐	☒	A full description of the statistical parameters including central tendency (e.g. means) or other basic estimates (e.g. regression coefficient) AND variation (e.g. standard deviation) or associated estimates of uncertainty (e.g. confidence intervals)
☐	☒	For null hypothesis testing, the test statistic (e.g. F, t, r) with confidence intervals, effect sizes, degrees of freedom and P value noted<br>*Give P values as exact values whenever suitable.*
☒	☐	For Bayesian analysis, information on the choice of priors and Markov chain Monte Carlo settings
☒	☐	For hierarchical and complex designs, identification of the appropriate level for tests and full reporting of outcomes
☒	☐	Estimates of effect sizes (e.g. Cohen's d, Pearson's r), indicating how they were calculated

Our web collection on statistics for biologists contains articles on many of the points above.

Software and code

Policy information about availability of computer code

Data collection	High-throughput image data were acquired on Opera Phenix microscope using Harmony High-Content Imaging and Analysis software (Perkin Elmer); super-resolution images were acquired on 3D-SIM OMX microscope (Delta Vision) using SoftWoRx software; laser micro irradiation data were collected on FluoView1000 confocal Olympus microscope and analysed using Fiji software.
Data analysis	For statistical analyses, Microsoft Excel (Microsoft Inc, USA) and GraphPad Prism 8 were used. Graphs were generated using GraphPad Prism 8. Images were processed in Fiji. Figures were prepared using Adobe Photoshop CS5 and Adobe Illustrator CS5 (Adobe Systems Inc, USA).

For manuscripts utilizing custom algorithms or software that are central to the research but not yet described in published literature, software must be made available to editors/reviewers. We strongly encourage code deposition in a community repository (e.g. GitHub). See the Nature Research guidelines for submitting code & software for further information.

Data

Policy information about availability of data

All manuscripts must include a data availability statement. This statement should provide the following information, where applicable:

- Accession codes, unique identifiers, or web links for publicly available datasets
- A list of figures that have associated raw data
- A description of any restrictions on data availability

All relevant data are available from the authors.

Field-specific reporting

Please select the one below that is the best fit for your research. If you are not sure, read the appropriate sections before making your selection.

☒ Life sciences ☐ Behavioural & social sciences ☐ Ecological, evolutionary & environmental sciences

For a reference copy of the document with all sections, see nature.com/documents/nr-reporting-summary-flat.pdf

Life sciences study design

All studies must disclose on these points even when the disclosure is negative.

Sample size	Sample size was not pre-determined.
Data exclusions	No data were excluded.
Replication	Most experiments were repeated at least 3 times, unless stated differently in figure legends. At least two independent clones of genetically modified (using CRIPR technology) cell lines were used for each assay. Sample size and number of independent experiments are stated in the figure legends.
Randomization	No randomization was applied
Blinding	No blinding was used during sample collection and processing. However, unless indicated, image acquisition and data analyses were performed in an unbiased way using high-throughput high-content Opera Phenix microscope and Harmony software.

Reporting for specific materials, systems and methods

We require information from authors about some types of materials, experimental systems and methods used in many studies. Here, indicate whether each material, system or method listed is relevant to your study. If you are not sure if a list item applies to your research, read the appropriate section before selecting a response.

Materials & experimental systems

n/a	Involved in the study
☐	☒ Antibodies
☐	☒ Eukaryotic cell lines
☒	☐ Palaeontology
☒	☐ Animals and other organisms
☒	☐ Human research participants
☒	☐ Clinical data

Methods

n/a	Involved in the study
☒	☐ ChIP-seq
☐	☒ Flow cytometry
☒	☐ MRI-based neuroimaging

Antibodies

Antibodies used

Antigen Raised in/type: poly/monoclonal Source, clone information Dilution for IB Dilution for IF
53BP1 Rabbit/poly Novus Biologicals, NB100-304 1:2000 1:500
ATM Rabbit/mono Abcam, [Y170], ab32420 1:2000
BRCA1 Mouse/mono Santa Cruz Biotechnology, D9 1:200 1:100
BRCA2 Mouse/mono Calbiochem/Merck, OP95 1:500
CtIP Mouse/mono Richard Baer, clone 14-1 1:50
Cyclin A Mouse/mono BD Biosciences, 611268 1:1000 1:100
GFP Mouse/mono Roche, 7.1+13.1 1:1000
GFP Rabbit/poly Life Technologies, 11122 1:1000
H2AX Rabbit/poly Abcam, ab11175 1:5000
H2B Rabbit/poly Abcam, ab1790 1:5000
H3 Rabbit/poly Abcam, ab1791 1:10000
H4 Rabbit/mono CST, 13919 1:1000
H2A Rabbit/poly Abcam, ab18255 1:1000
6xHis mouse/mono Genscript A00186 1:20000
MBP mouse/mono NEB/ E8032S 1:50000
MBP Rabbit/poly Sigma Aldrich, MBP-17 1:50000
MRG15 Rabbit/mono Cell Signaling, D2Y4J, 14098 1:1000
NBS1 Rabbit/poly Abcam, ab23996 1:1000
PALB2 Rabbit/poly Gift of Bing Xia, 25
1:500 1:100
RAD50 Mouse/mono Abcam, [13B3/2C6], ab89 1:1000
RAD51 Rabbit/poly Santa Cruz Biotechnology, H-92 1:500 1:100

	RNF168 Rabbit/poly Millipore, ABE367 1:1000<br>RNF169 Rabbit/poly Abcam, ab87711 1:1000<br>RPA32 Mouse/mono Abcam, [9H8], ab2175 1:250<br>RPA32 Mouse/mono Mouse hybridoma, Shiloh lab 1:20<br>-tubulin Mouse/mono Sigma Aldrich, T9026 1:104
Validation	Each experiment had appropriate controls to validate the antibodies. Commercially available antibodies were validated by the supplier and by us using appropriate controls where needed; Supplementary Table 2. Please also refer to the manufacturers' websites for further details.

Eukaryotic cell lines

Policy information about cell lines

Cell line source(s)	U2OS(TREX), U2OS(TLR), RPE1 (FRT), RPE1p53-(FRT), HEK293T: SP Jackson (Gurdon Institute, Cambridge, UK)
Authentication	All cells were originally obtained from the ATCC cell repository, and we have authenticated cell lines used in our study by STR profiling.
Mycoplasma contamination	All cell lines are routinely tested to be mycoplasma-free
Commonly misidentified lines<br>(See ICLAC register)	No commonly misidentified cell lines were used

Flow Cytometry

Plots

Confirm that:

- [ ] The axis labels state the marker and fluorochrome used (e.g. CD4-FITC).
- [ ] The axis scales are clearly visible. Include numbers along axes only for bottom left plot of group (a 'group' is an analysis of identical markers).
- [ ] All plots are contour plots with outliers or pseudocolor plots.
- [ ] A numerical value for number of cells or percentage (with statistics) is provided.

Methodology

Sample preparation	U2OS(TLR) cells (based on Certo et al. 2011 Nat Methods, Jul 10;8(8):671-6) were transfected with the indicated siRNAs and 6-8 h later cells were co-transfected with the HR-Donor and I-SceI expression plasmids. After ~72 h, cells were trypsinised and collected in 1% PBS/BSA.
Instrument	BD LSRFortessa cell analyser (BD Biosciences)
Software	FlowJo(BD Inc, USA)
Cell population abundance	Cell sorting was not necessary to evaluate homologous recombination events, and was not performed for this assay.
Gating strategy	FSC/SSC gates define single cell population. For each condition, 10,000 live cells which were successfully expressing donor (BFP) and I-SCeI(IFP) were scored for GFP (HR) and mCherry (mutEJ). GFP and mCherry gates were defined using BFP/IFP negative cell population as a negative control. Each siRNA treatment was normalised to a negative control siRNA targeted to firefly luciferase.

- [ ] Tick this box to confirm that a figure exemplifying the gating strategy is provided in the Supplementary Information.
