# Supplementary material for: PALB2 chromatin recruitment restores homologous recombination in BRCA1-deficient cells depleted of 53BP1
Source: Nat Commun. 2020 Feb 10;11:819. doi: 10.1038/s41467-020-14563-y (PMC7010753; doi:10.1038/s41467-020-14563-y)

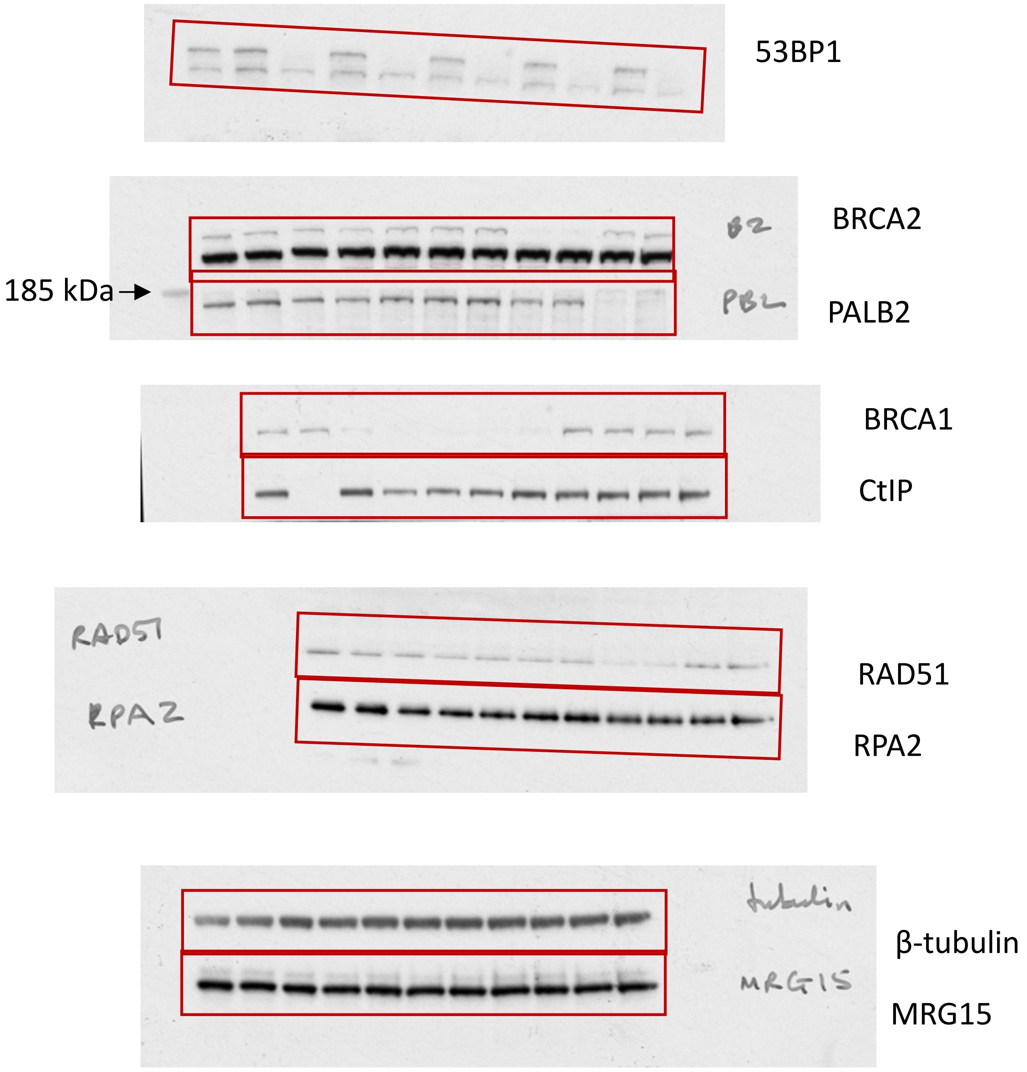
53BP1
B2
BRCA2
185 kDa
PBL
PALB2
BRCA1
CtIP
RAD51
RPA2
RAD51
RPA2
tubulin
β-tubulin
MRG15
MRG15
Fig. 1b

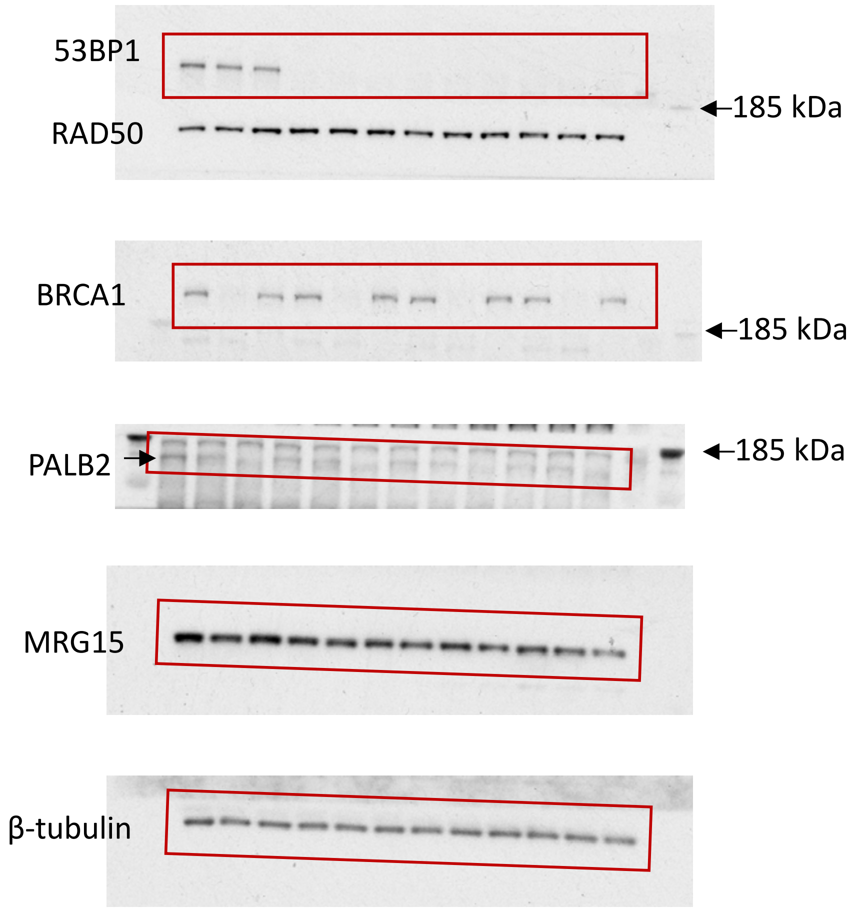
53BP1
RAD50
185 kDa
BRCA1
185 kDa
PALB2
185 kDa
MRG15
β-tubulin
Fig. 1e

Supplementary Fig. 1b

185 kDa →

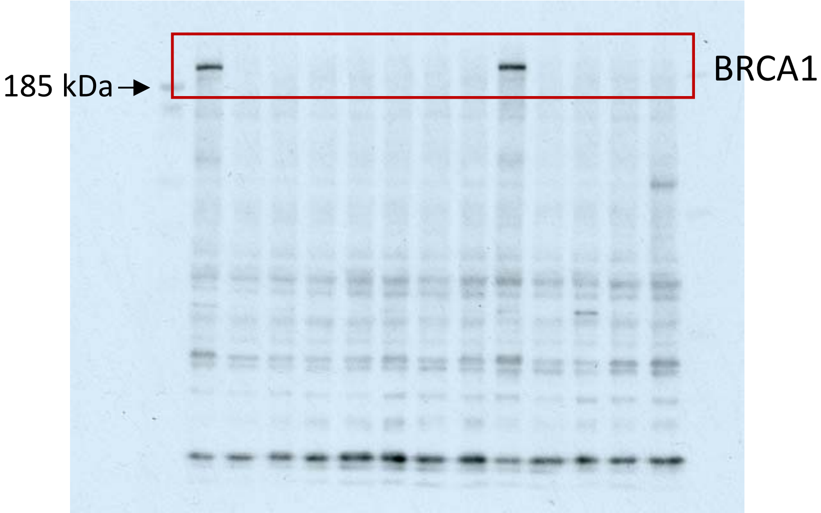

BRCA1

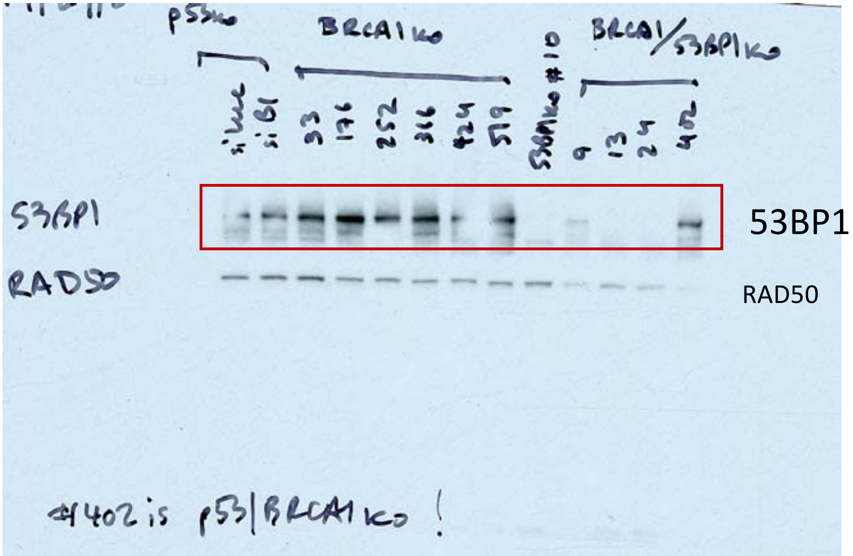

53BP1

RAD50

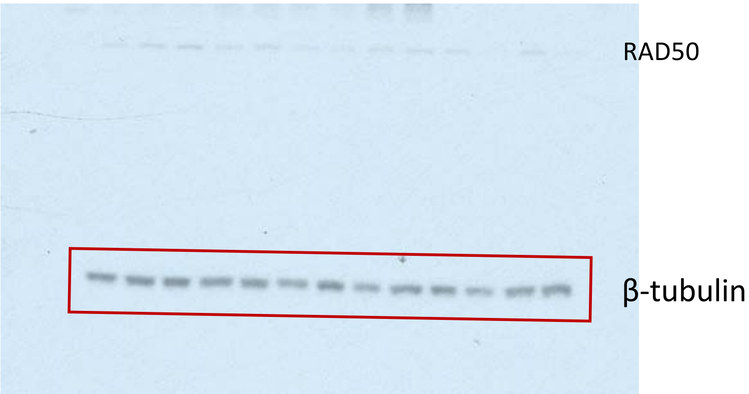

RAD50

β-tubulin

Supplementary Fig. 2

h

f

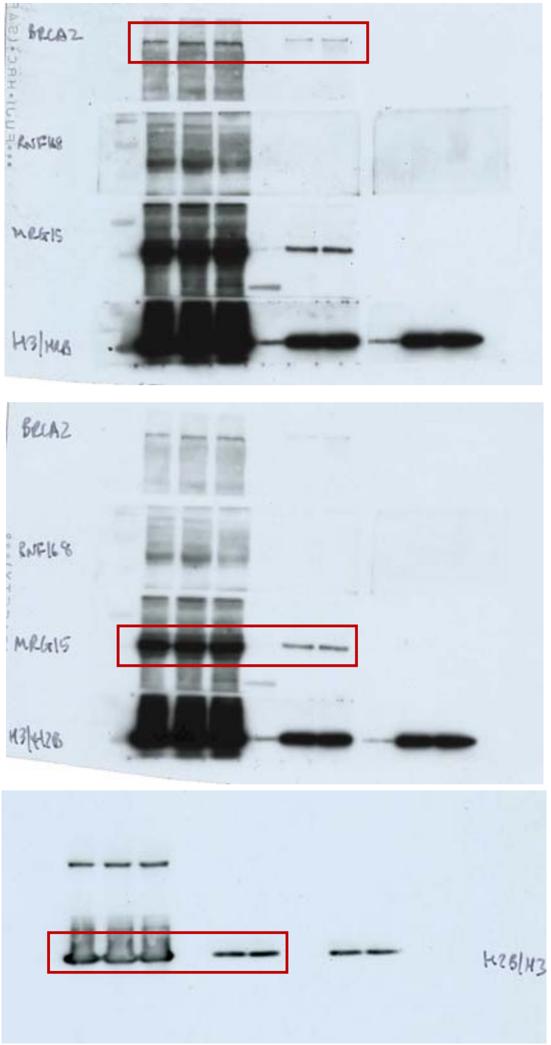

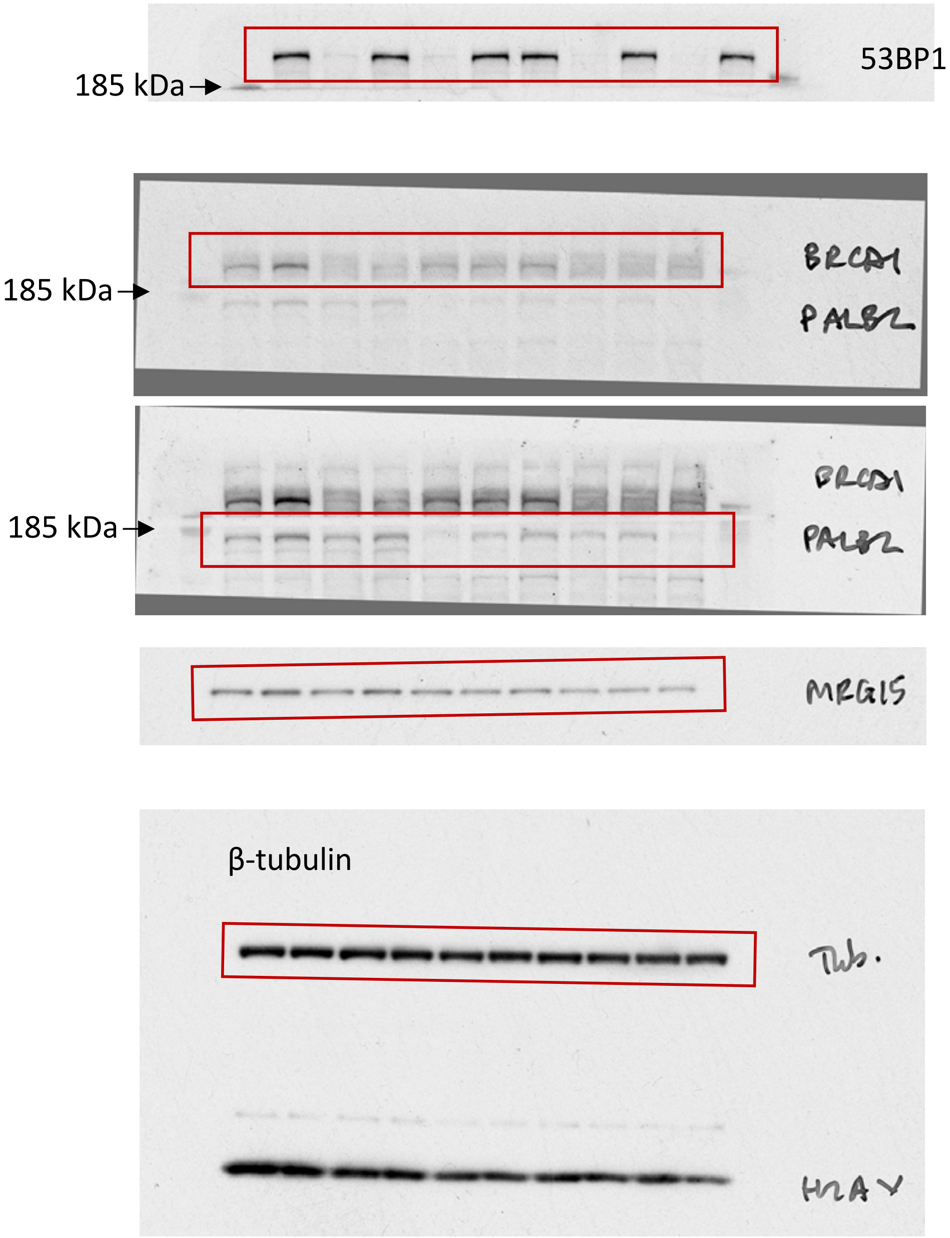

Supplementary Fig. 4c

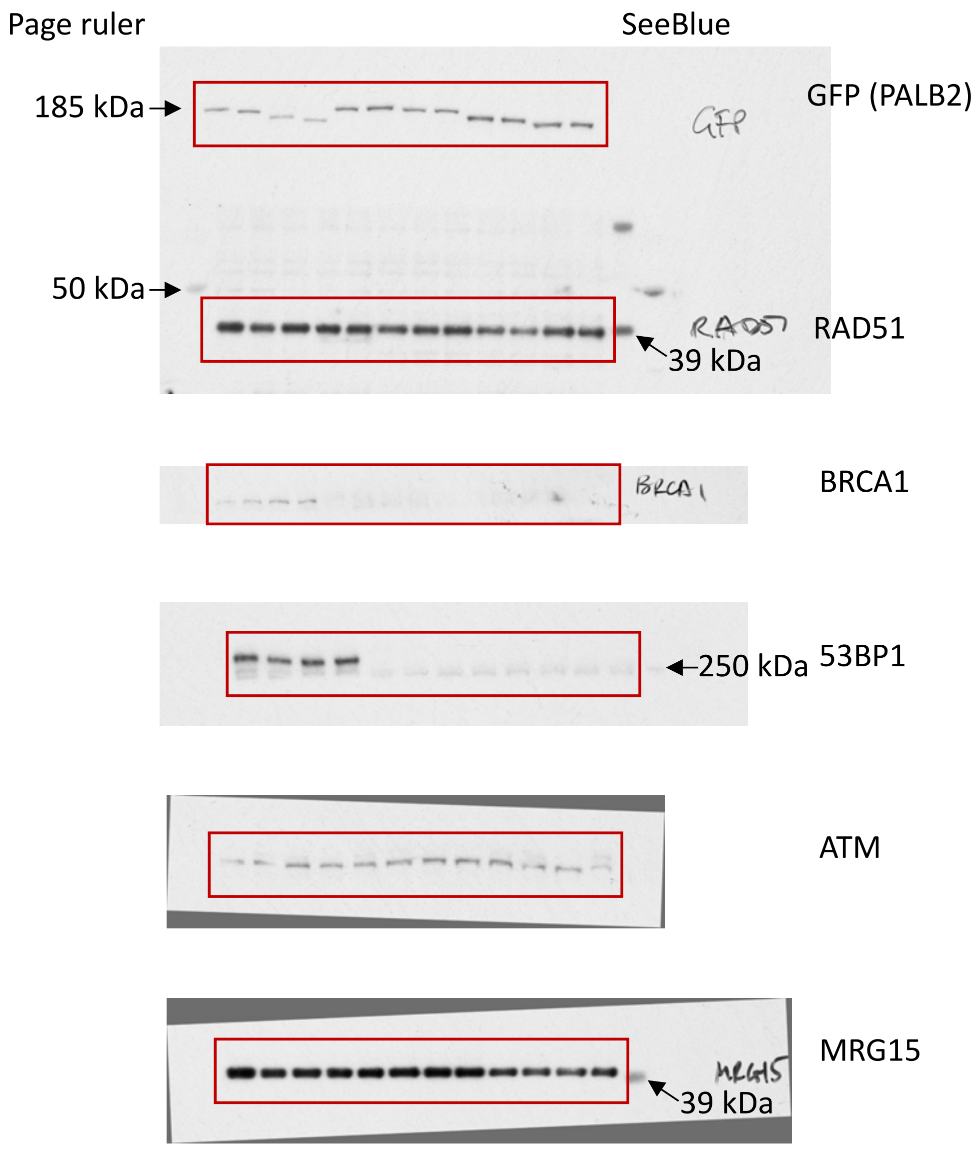

Fig. 4

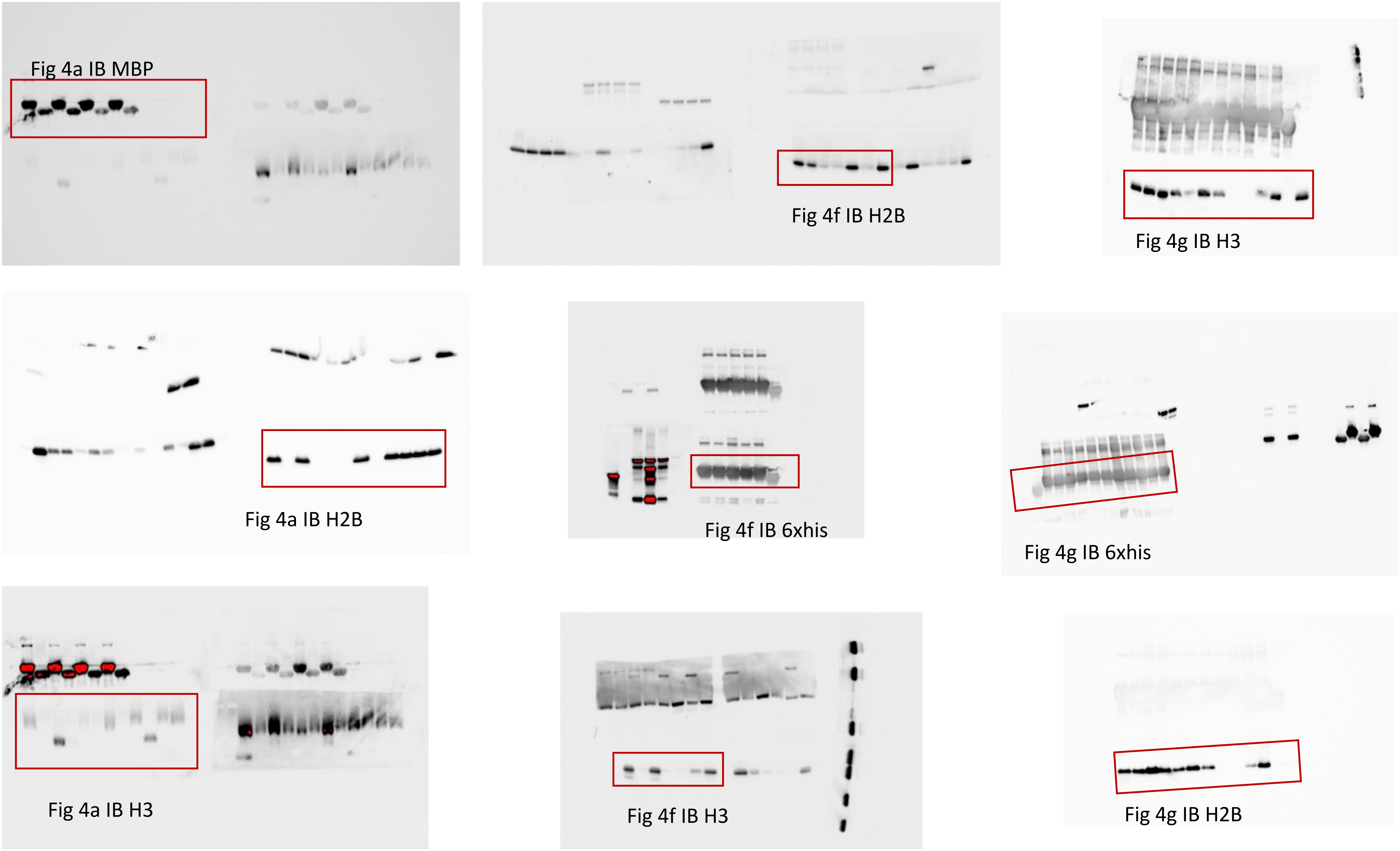
Fig 4a IB MBP
Fig 4f IB H2B
Fig 4g IB H3
Fig 4a IB H2B
Fig 4f IB 6xhis
Fig 4g IB 6xhis
Fig 4a IB H3
Fig 4f IB H3
Fig 4g IB H2B

Supplementary Fig. 5

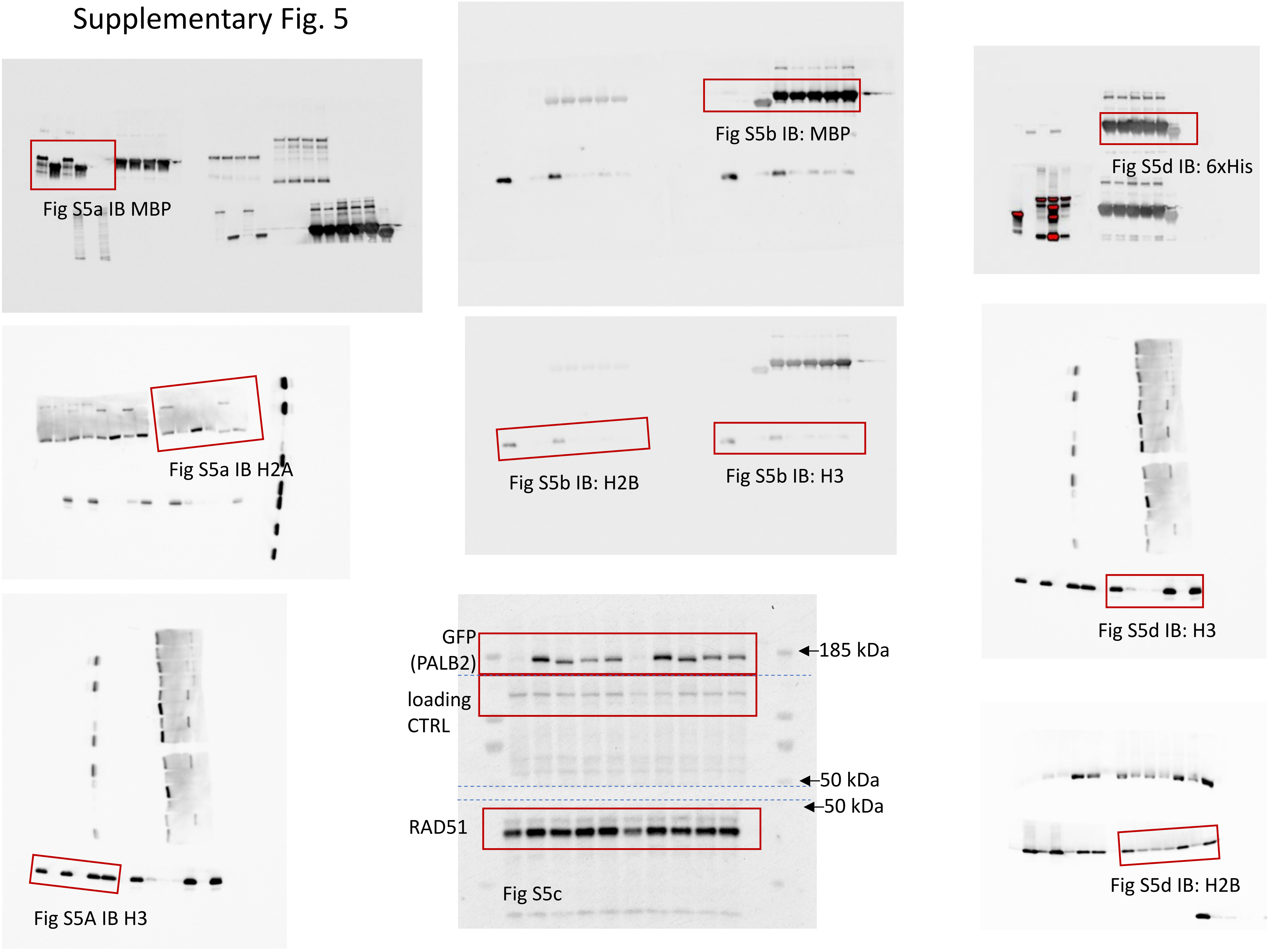

Supplementary Fig. 6c

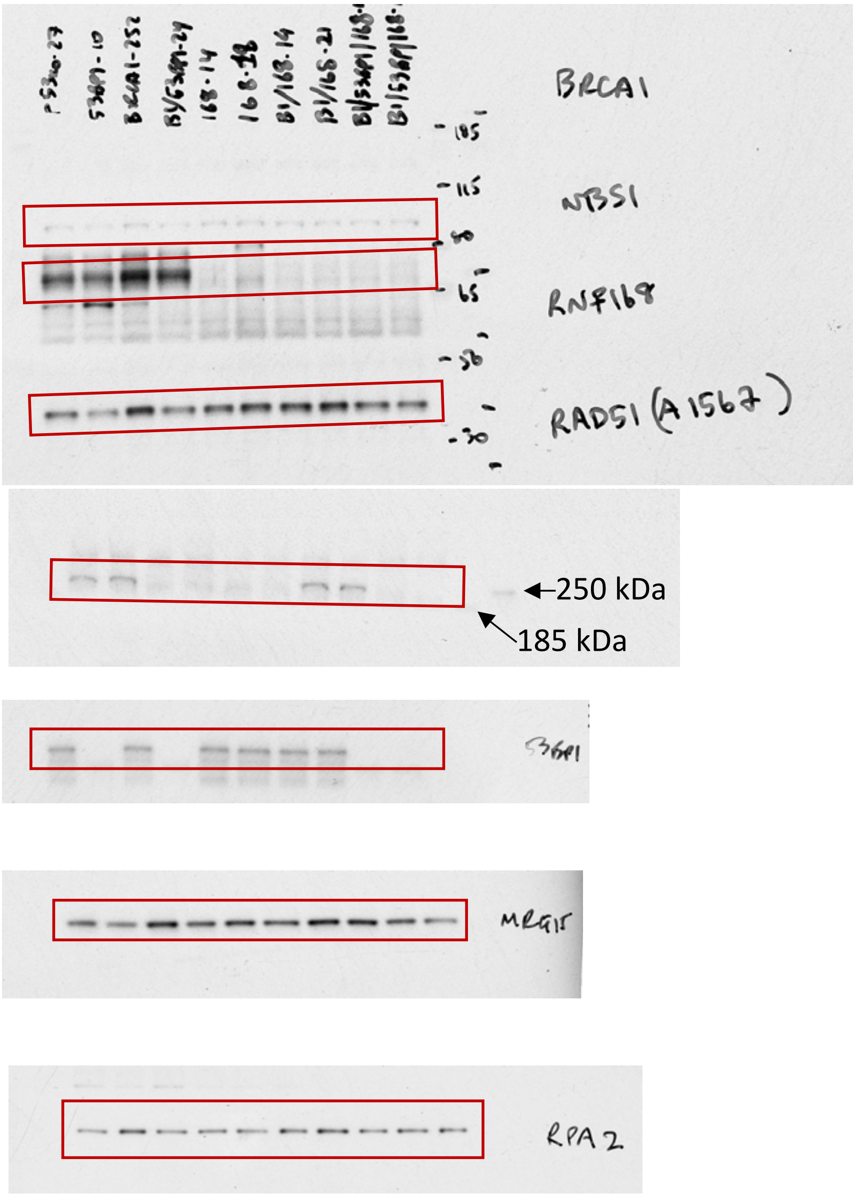

Supplement: Supplementary file 8 — Source Data [file 41467_2020_14563_MOESM8_ESM.zip › sd2.pdf]
